# Corruption: possibly the biggest threat to health care

**DOI:** 10.1016/j.lana.2024.100744

**Published:** 2024-04-10

**Authors:** The Lancet Regional Health – Americas

Political corruption is in the news every day. Corruption can happen in any country, any level, regardless of political views, gender, or even social status, and it has been documented for millennia. It is estimated that the money lost to corruption globally surpasses US$3.5 trillion per year. However, corruption not only means economic personal gain, but also immoral actions. In addition, political corruption spills over health care administration, which is very concerning as the health of people does not only depend on direct medical care; it is also in the hands of political and health care authorities.

Probably one of the biggest healthcare-related scandals in the history of Peru is linked to the COVID-19 pandemic, which has killed more than 200,000 Peruvians. Millions of people were expecting the arrival of the first lot of Sinopharm vaccines in Peru at the beginning of 2021. Earlier, in the second half of 2020, a clinical trial in Peru was ongoing to test the efficacy of the Sinopharm vaccine. On February 7, 2021, the first lot of vaccines arrived. According to Government officials, health-care workers on the frontline working in intensive care units and emergency rooms would be the first to receive the vaccine. However, hundreds of people who were not participants in the clinical trial, many of them influential, had secretly received the vaccine the year before. This big scandal was known as vacunagate, a shameful, unethical, and corrupt action committed by 487 people including some influential Peruvian scientists, public health authorities, politicians—among them, the former president of Peru, Martín Vizcarra—businesspeople, and their relatives. The shocking news was officially released on February 14, 2021, and the list of people involved was released 2 days later. It has been 3 years since the vacunagate scandal went public. However, vacunagate is still an ignominy to millions of Peruvians who saw how their relatives, friends, and colleagues succumbed to the virus.

The Peruvian Congress witnessed unconvincing responses from the leader of the clinical trial, who ultimately decided who would deserve to be vaccinated secretly. During that time, those in the Government, former members of the Government, and also high authorities of universities, received not one, but two or three doses, before even health-care professionals caring for critically ill patients could have access to the first dose. More than 720 physicians and nurses died because of COVID-19 during the pandemic. Vacunagate is a clear example of how power and influence can bypass the most fundamental ethical principles, even during a global emergency. It fully displays corruption at the highest level in health care.

Big corruption scandals in health care are not uncommon in the region. Health-care authorities of Brazil, particularly in Rio de Janeiro, have been subjected to close scrutiny because of corruption related to bribery and personal benefits from the budget that was supposed to be destined for health-care services for millions of Brazilians. It was estimated that US$350 million was diverted to murky contracts since 2007. It is difficult to measure the magnitude of the direct effect of corruption on health care, but there are certainly important consequences, from shortage of health workers to disruption of medical attention, infrastructure expansion, and maintenance of medical equipment. The list of corruption stories across the Americas is long. And knowing that corruption is a ubiquitous phenomenon, it tells us that more action is needed.

In March 2024, the Consortium of Universities on Global Health held its 15th annual conference in Los Angeles, CA, USA. The most pressing global health issues were addressed, including two sessions on corruption in health care. Despite there being awareness around the issue, there are few action points. According to global health experts, the health-care crisis in some African countries, for example, might be due to corruption, not absence of money. Indeed, data from 133 countries show that higher corruption is associated with lower levels of health expenditure and poorer health outcomes. In September, 2023, WHO released a policy brief to tackle corruption as a way to achieve universal health care. Corruption undermines the whole health-care system. The question is how to effectively fight corruption if cases like those in Peru and Brazil show that top government authorities themselves are implicated in corruption. According to WHO, in some countries, up to 80% of health budget never reaches its purpose, although exact data are not available. WHO proposes that anti-corruption measures, transparency, and accountability should be incorporated into health systems strategies to tackle this public health issue and should focus primarily on prevention.

While prevention measures are certainly needed to tackle corruption, if confirmed, impunity should not be the norm. The example of corruption in Rio de Janeiro resulted in the impeachment of its Governor, but not all cases of corruption end in a trial process. For unethical incidents, like what occurred in Peru, apologies to the nation would be better than unconvincing excuses. Identifying suspected acts of corruption requires collaboration at different levels, from justice authorities to civil society, including journalists and whistle-blowers. Countries with solid health-care administrations, if not affected by corruption, could have healthier nations, with longer life expectancy. However, indifference to corruption leads to severe disruptions in health care at all levels, with vulnerable people being the most affected. Experts say we need a strong culture of integrity to tackle corruption. The challenge is to change structural unethical issues, and we can start by reinforcing the education of values in the younger generations.

